# Is the third molar the most frequently extracted tooth? A population-based study utilizing dental panoramic radiographs in adults

**DOI:** 10.1007/s00784-024-05845-6

**Published:** 2024-07-24

**Authors:** Irja Ventä, Johanna Snäll, David P Rice, Anna Liisa Suominen

**Affiliations:** 1 Department of Oral and Maxillofacial Diseases, Faculty of Medicine, University of Helsinki, Helsinki, Finland; 2 Department of Oral and Maxillofacial Diseases, Helsinki University Hospital, Helsinki, Finland; 3 Institute of Dentistry, University of Eastern Finland, Kuopio, Finland; 4 Oral and Maxillofacial Teaching Unit, Kuopio University Hospital, Kuopio, Finland; 5 Public Health Evaluation and Projection Unit, The Finnish Institute for Health and Welfare, Helsinki, Finland

**Keywords:** Third molar, Panoramic radiography, Tooth extraction, Adults, Population characteristics

## Abstract

**Objectives:**

The study aimed to examine the authenticity of the often-mentioned statement that the third molar is the most frequently extracted tooth. This finding has not been shown previously in a large population-based sample.

**Materials and methods:**

Data comprised a nationally representative sample of 6082 panoramic radiographs taken from adults in the cross-sectional Health 2000 Survey. From the radiographs, all missing teeth were recorded. Information on congenital agenesis of individual teeth was retrieved from two published meta-analyses. Primary outcome was the frequency of missing teeth by tooth type. Explanatory variables were age, sex, and the jaw (maxilla/mandible). Statistical analyses included χ^2^ test and binomial logistic regression.

**Results:**

Mean age of participants (46% men, 54% women) was 53 years (SD 14.6; range 30‒97 years). Missing teeth occurred more often in women than in men (*P* < 0.001). The third molar was most frequently missing and the canine least frequently. In the maxilla and mandible, the third molar was missing more often than each of the other tooth types up to the age of 80 years (*P* < 0.01).

**Conclusions:**

When considering the rates of congenital agenesis of individual teeth, it is concluded that the third molar remained the most common tooth extracted up till the age of 80 years.

**Clinical relevance:**

The third molar is the most common target for extraction, but also the most common tooth associated with malpractice claims, and therefore, calls for skills, adequate equipment, and other resources for a successful extraction.

## Introduction

Several scientific articles on third molars begin with the statement that the third molar is the most frequently extracted tooth [1–7]. Also, it is often stated that extraction of this tooth is the most common surgical procedure in dentistry. References are typically not given for these statements. Clinically, it is obvious that third molar extraction is the most frequent type of treatment at oral surgical units and in young adults. However, this topic has not been commonly analyzed using population-level data on all teeth on the grounds of the most frequently extracted or missing tooth. The lack of scientific evidence is mainly due to two reasons; third molars are often ignored in clinical oral examinations of large populations [8, 9] and panoramic radiographs are rarely obtained in population studies [8–11].

According to clinical oral examinations of several distinguished population studies, mandibular incisors and canines are the most long-lasting teeth [8–12]. More detailed information about individual teeth is available on the prevalence of third molars based on two larger studies with panoramic radiographs. In a nationwide survey of Finnish adults, all four third molars are missing in the age group 30‒39 years in 37.5% of individuals and in persons older than 70 years in 80.8% [13]. A similar trend in missing third molars has been reported in a Swedish radiographic study [14]. However, there are no population-level studies on missing teeth based on radiological findings and evaluating the entire dentition.

When assessing the rates of extracted or missing teeth at population level, congenital absence of teeth must be considered. In a meta-analysis on the agenesis of third molars, the worldwide rate for at least one congenitally missing third molar is 22.6% of individuals and for all four third molars 3.4% [15]. According to another meta-analysis on teeth other than third molars, prevalence of agenesis of one or more teeth varies between 2.2% and 7.7% depending on the continent, race, and sex [16]. Of single teeth, the mandibular second premolar is the most frequent absent tooth at 2.9–3.2%, followed by upper lateral incisor at 1.6–1.8% and upper second premolar at 1.4–1.6%. In individuals with tooth agenesis, one or two absent teeth is far more common (82.9%) than four missing teeth (6.0%) [16].

The aim of this study was to examine which tooth is most frequently missing from panoramic radiographs when evaluating the entire dentition of a nationally representative adult population. From the analysis of missing teeth, it is possible to conclude the most frequently extracted tooth. Based on earlier prevalence studies, the hypothesis was that the third molar is the most frequently extracted tooth.

## Materials and methods

### Study design

This study was part of the Health 2000 Survey (BRIF8901, Bioresource Research Impact Factor), which was a cross-sectional study organized in 2000 − 2001 by the Finnish Institute for Health and Welfare (THL) [17, 18]. The main sample of the nationally representative survey consisted of 8028 adults aged 30 years and older. The present analysis was based on panoramic radiographs, which were obtained after the clinical oral examination.

The research protocol included a two-stage stratified cluster sampling strategy, calibration of radiologists, diagnostic quality control in the examination of radiographs, and missing data analysis [12, 18, 19]. The sampling design covered all five university hospital regions as stratums and 80 health center districts as clusters from which the target persons were selected using systematic random sampling [17, 18]. The radiologists were trained beforehand by examining 50 radiographs, whereby diagnostic criteria were specified [12]. When examining the radiographs, the same radiologist re-examined an earlier image taken a day before or earlier, for every 30th radiograph [12]. The participants in the clinical oral examination did not differ by sex but were younger than the nonparticipants [19].

From the starting sample, 6335 individuals participated in the clinical oral examination, and digital panoramic radiographs were obtained from 6115 participants. All radiographs were evaluated by specialists in oral radiology. Due to insufficient quality of radiographs, 14 participants were excluded. Data on 19 participants were not available for the present examination due to withdrawal from the study, and thus, the final sample comprised 6082 participants.

### Study variables

Data on participants’ age and sex were retrieved from the Population Register of Finland. The location and condition of each tooth were recorded from the radiographs as follows: missing, impacted, root remains, retained root in bone, dental implant, carious tooth, restored or sound tooth. Number of each participant’s missing teeth, including implants, was calculated from the data.

The primary outcome was the frequency of missing teeth by tooth type. Explanatory variables were age, sex, and the jaw (maxilla or mandible).

### Statistical analysis

Differences in frequencies between categorical variables were tested with χ^2^ test. Analyses were performed with SPSS Statistics, version 29 (IBM Corporation, Armonk, NY, USA). Age of participants was grouped as 30‒39, 40‒49, 50‒59, 60‒69, 70‒79, 80‒89, and ≥ 90 years. Teeth were categorized by tooth types as central incisors, lateral incisors, canines, first premolars, second premolars, first molars, second molars, and third molars. Numbers of missing teeth per participant were calculated, and comparisons were made between the upper and lower jaw. Differences between sexes were assessed. Rates of missing teeth by tooth types were analyzed according to age groups separately for the maxilla and the mandible.

A binomial logistic regression was performed on each tooth type in the maxilla and in the mandible to ascertain the associations of age and sex of participants with the likelihood of missing teeth. Odds ratios (ORs) and their 95% confidence intervals (CIs) were reported. Finally, an estimation of truly missing teeth was made based on percentages of congenital agenesis of teeth according to information from the literature [15, 16].

### Ethical considerations

This study followed the Declaration of Helsinki on medical research protocols and ethics. All participants signed a written informed consent before the clinical and radiographic examination. For clinical examinations, ethical approval was received from two committees: the Ethics Committee of the National Public Health Institute (KTL1999_6167) and the Ethics Committee of Epidemiology and National Health in the Hospital District of Helsinki and Uusimaa (TET3/9/310,500). For radiography, a safety license was granted by the Radiation and Nuclear Safety Authority of Finland (4969/L1/00). Permission for the present investigation was obtained from the THL Biobank (THLBB2023_13).

## Results

Of the 6082 included participants with panoramic radiographs, 46% were men and 54% women (Table 1). The mean age of participants was 53 years (SD 14.6; median 51; range 30‒97 years). Full dentition with 32 teeth occurred in 41% (*n* = 2469) of participants, and 12% (*n* = 739) were fully edentulous.

**Table 1:** Description of the 6082 participants classified according to age and sex

Age (years)	Men		Women		Both combined	
	n	(%)	n	(%)	n	(%)
30–39	658	(23.6)	690	(21.0)	1348	(22.2)
40–49	711	(25.4)	790	(24.0)	1501	(24.7)
50–59	654	(23.4)	699	(21.3)	1353	(22.2)
60–69	426	(15.3)	508	(15.4)	934	(15.3)
70–79	234	(8.4)	366	(11.1)	600	(9.9)
80–89	101	(3.6)	227	(6.9)	328	(5.4)
≥ 90	9	(0.3)	9	(0.3)	18	(0.3)
Total	2793		3289		6082	

In the analysis of all participants and all tooth types, the third molar was the most frequently missing and the canine the least frequently (Table 2). As regards all four teeth missing, significant differences at *P* < 0.001 were observed in frequencies between the third molar and each of the other tooth types.

**Table 2:** Distribution of missing teeth by tooth type in 6082 persons

Tooth type	Number of missing teeth, *n* (%)					
	0	1	2	3	4	At least 1
Central incisor	4141 (68)	168 (3)	699 (11)	65 (1)	1009 (17)	1941 (32)
Lateral incisor	3937 (65)	320 (5)	760 (13)	94 (1)	971 (16)	2145 (35)
Canine	4171 (69)	254 (4)	630 (10)	147 (2)	880 (15)	1911 (31)
1st premolar	3334 (55)	612 (10)	704 (12)	314 (5)	1118 (18)	2748 (45)
2nd premolar	2787 (46)	809 (13)	683 (11)	452 (8)	1351 (22)	3295 (54)
1st molar	2298 (38)	741 (12)	752 (12)	545 (9)	1746 (29)	3784 (62)
2nd molar	2606 (43)	676 (11)	584 (10)	498 (8)	1718 (28)	3476 (57)
3rd molar	473 (8)	449 (7)	871 (14)	1085 (18)	3204 (53)	5609 (92)

Teeth were missing more often in the maxilla than in the mandible in all tooth types, except for the first and second molars, where the mandible prevailed (*P* < 0.001 for all tooth types). In both the maxilla and the mandible, the third molar showed the highest rates of missing teeth, 79% and 71%, respectively.

Each of the eight tooth types were missing more often in women than in men (*P* < 0.001) (Table 3). In both sexes, the third molar was the most frequently missing tooth type. The greatest proportional difference between the sexes occurred in third molars; all four were missing in 60% of women and in 44% of men (χ^2^ = 165.14; df = 1; *P* < 0.001).

**Table 3:** Differences between sexes in numbers of missing teeth by tooth type in 6082 persons

Tooth type	Men				Women			*P* ^a^
	Number of missing teeth, n (%)							
	0	1‒3	4		0	1‒3	4	
Central incisor	1923 (69)	480 (17)	390 (14)		2218 (67)	452 (14)	619 (19)	< 0.001
Lateral incisor	1841 (66)	582 (21)	370 (13)		2096 (64)	592 (18)	601 (18)	< 0.001
Canine	1980 (71)	487 (17)	326 (12)		2191 (67)	544 (16)	554 (17)	< 0.001
1st premolar	1582 (57)	780 (28)	431 (15)		1752 (53)	850 (26)	687 (21)	< 0.001
2nd premolar	1314 (47)	938 (34)	541 (19)		1473 (45)	1006 (30)	810 (25)	< 0.001
1st molar	1051 (38)	1010 (36)	732 (26)		1247 (38)	1028 (31)	1014 (31)	< 0.001
2nd molar	1224 (44)	858 (31)	711 (25)		1382 (42)	900 (27)	1007 (31)	< 0.001
3rd molar	296 (10)	1275 (46)	1222 (44)		177 (6)	1130 (34)	1982 (60)	< 0.001

^a^Chi-squared test between sexes; df = 2

**Fig. 1:**
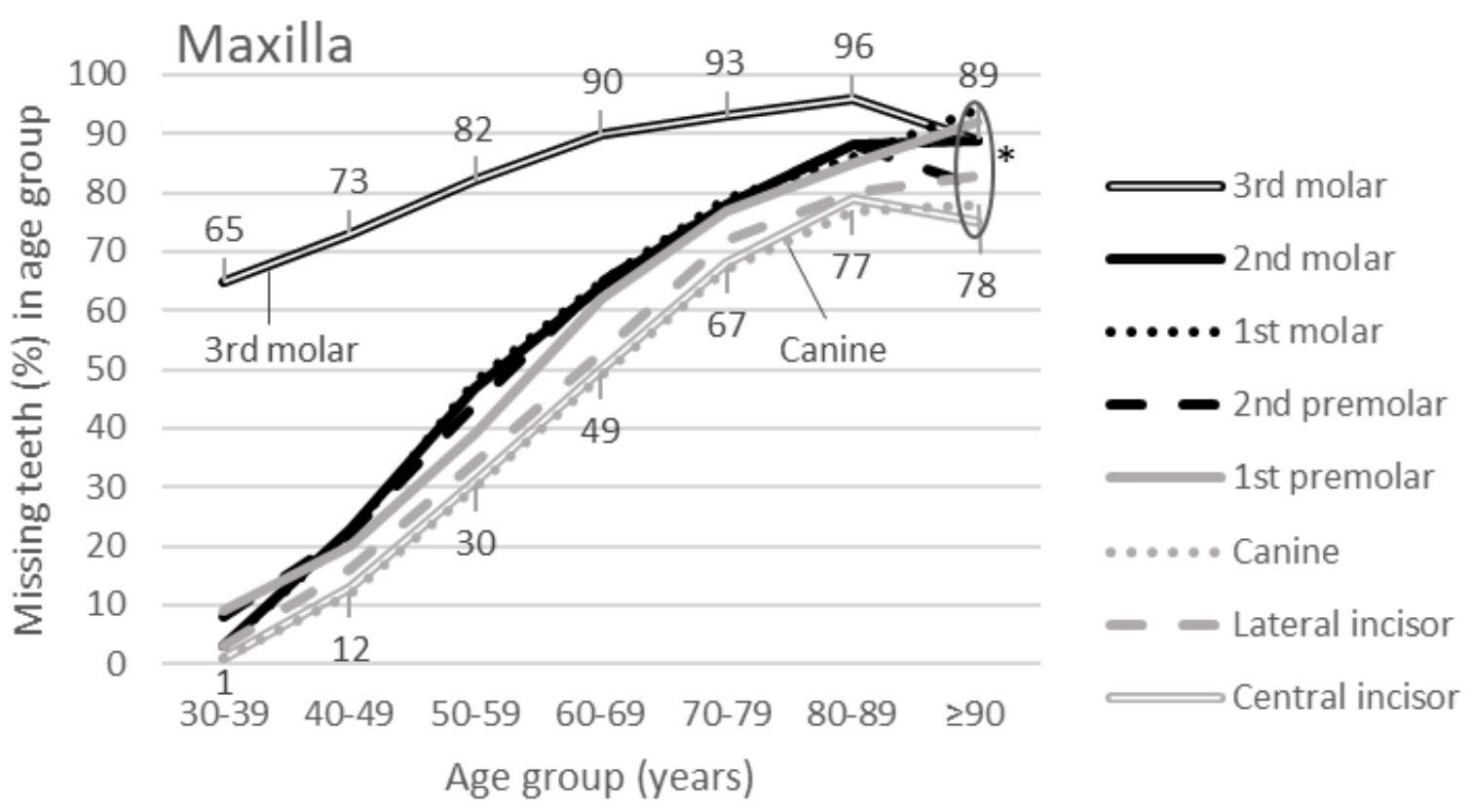
Percentage curves of missing teeth by tooth type in the maxilla according to age group (*N* = 6082 persons). Percentage values are shown only for the third molar and canine (highest and lowest curves). Asterisk (*) shows comparisons where a statistically significant difference in missing teeth was not found between the third molar and all other tooth types

Occurrence of missing teeth according to age group is presented in Fig. 1 (maxilla) and 2 (mandible). In both jaws, the third molar was missing most often and the canine or incisors least frequently. A significant difference at *P* < 0.01 was found in all comparisons between the frequencies of the third molar and each of the other tooth types, except in the two oldest age groups.

**Fig. 2:**
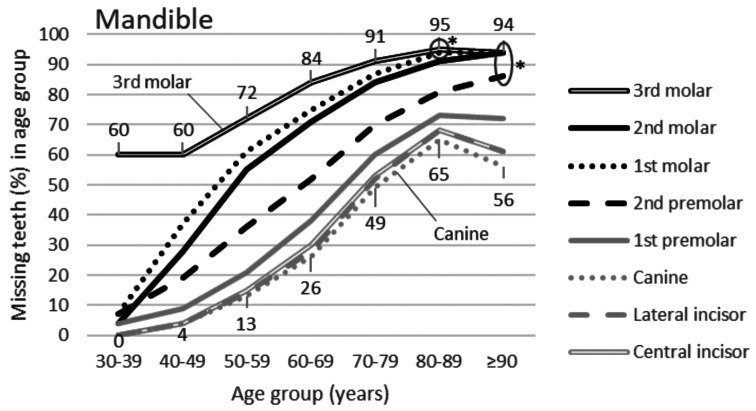
Percentage curves of missing teeth by tooth type in the mandible according to age group (*N* = 6082 persons). Percentage values are shown for the third molar and canine. Two asterisks (*) show comparisons where statistically significant differences in missing teeth were not found between the third molar and some other tooth types

In the logistic regression analysis, a significant covariate (*P* < 0.001) for all tooth types to explain the absence of teeth was age; the higher the age the more likely a tooth was missing. Regarding sex, both in the maxilla and the mandible, third molars were twice as likely to be missing in women than in men (OR for the maxilla 2.0; 95% CI 1.75‒2.40 and the mandible 1.8; 95% CI 1.59‒2.09). The prevailing sex was reversed for the upper first molar, as it was 1.3 times (95% CI 1.18‒1.52) more likely missing in men than in women. The logistic regression models for each tooth type were significant at *P* < 0.001 (Omnibus tests; df = 2), and correctly classified 73‒87% of cases.

Considering the rates of congenital agenesis of individual teeth, the third molar remained the most frequently missing tooth (Table 4). The second most commonly missing tooth type was the first molar in the mandible. The sequence of other teeth is present in Table 4.

**Table 4:** Comparison between present results on rates of missing teeth and congenital agenesis of individual teeth derived from meta-analyses in the literature [15, 16]. Final column shows the rank of the most commonly missing teeth

Jaw and tooth type		Present result % (95% CI)	Agenesis in literature % (95% CI)	Difference^a^ %	Rank
Maxilla					
	Central incisor	29 (28.69‒30.31)	0.00‒0.01	28.68	10.
	Lateral incisor	32 (30.94‒32.59)	1.55‒1.78	29.16	9.
	Canine	29 (27.74‒29.35)	0.07‒0.13	27.61	11.
	1st premolar	37 (36.39‒38.11)	0.17‒0.25	36.14	7.
	2nd premolar	40 (38.75‒40.49)	1.39‒1.61	37.14	6.
	1st molar	40 (38.74‒40.48)	0.02‒0.05	38.69	4.
	2nd molar	39 (38.55‒40.29)	0.03‒0.06	38.49	5.
Mandible					
	Central incisor	18 (17.48‒18.85)	0.25‒0.35	17.13	13.
	Lateral incisor	18 (16.93‒18.29)	0.17‒0.25	16.68	14.
	Canine	16 (15.81‒17.13)	0.01‒0.03	15.78	15.
	1st premolar	24 (23.11‒24.62)	0.10‒0.17	22.94	12.
	2nd premolar	34 (33.00‒34.68)	2.91‒3.22	29.78	8.
	1st molar	50 (48.82‒50.59)	0.00‒0.02	48.80	2.
	2nd molar	45 (43.63‒45.40)	0.07‒0.13	43.50	3.
Both jaws					
	3rd molar				1.
	At least one	92 (91.55‒92.90)	21.60 (18.4‒25.1)^b^	66.45	
	All four	53 (51.43‒53.93)	3.42 (2.9‒4.0)	47.43	

^a^Difference: Present result (lowest value) minus agenesis in literature (highest value)

^b^Figures for Europeans

CI: confidence interval

## Discussion

The purpose of the study was to establish the authenticity of the often-mentioned statement that the third molar is the most frequently extracted tooth. The findings confirmed the hypothesis; among all tooth types, the third molar was the most frequently extracted tooth. However, this preponderance began to taper off after the age of 80 years. In consideration of the rates of congenital agenesis of teeth, the third molar remained the most extracted tooth.

Based on this large nationwide population data with panoramic radiographs, the most important finding was that the third molar was the most extracted tooth. This finding has not been shown previously in a large population-based sample. The sample of the Health 2000 Survey was collected using a two-stage stratified cluster sampling strategy to properly represent the main demographic distribution of the Finnish population [12, 17–19]. Also, the rate of participation in the survey was very high; of the initial sample of 8028 adults, 79% participated in the clinical oral examination and 76% in radiography [12, 17–19]. Thus, the sample was suitable for the purpose of the study.

Another interesting finding was that there was an age limit for the third molar being the most frequently extracted tooth type; in the maxilla, it was 90 years for all tooth types, and in the mandible, the limit was 80 years for the first molar and 90 years for the other three posterior teeth. No age limit emerged for anterior teeth in the mandible. The age limit is a new finding. This limit is explained by the progressive loss of other teeth catching up with the level of the third molar. Tooth extractions in a general population typically accumulate in young adults and in persons older than 75 years [20]; especially the third molar prevails in young adults [2, 21, 22]. However, the present cross-sectional analysis showed that the preponderance of third molars among all missing teeth occurred until the age of 80 years.

In the bivariate analysis, each of the eight tooth types was missing more often in women than in men. Particularly the third molar was more than twice as likely to be missing in women than in men. This preponderance of women with missing third molars has also been shown earlier [14, 22]. Women are ahead of men regarding congenital agenesis of teeth, as well [15, 16]. According to the referred meta-analyses, congenital agenesis of teeth other than third molars is 1.37 times higher in women than in men [16], and at least one missing third molar is 14% more likely to occur in women than in men [15]. This finding may also suggest that women visit the dentist more regularly, and thus, more often undergo extractions.

A comparison of the present findings with those in the literature is difficult, as large population studies with panoramic radiographs are relatively rare. However, a US study with a large number of insured patients, states that third molars are the most extracted permanent teeth [21]. In more restricted samples, such as dental specialties, the third molar may not be the most frequently extracted tooth, for example, in orthodontics, it is not uncommon to extract premolars [23]. In general, studies on presence of teeth do not even include third molars, nevertheless, they show that teeth are lost along with increasing age and posterior teeth more frequently than anterior teeth [8–11, 24]. Similar illustrations of tooth loss by age group as in the present study are also reported from Greece for some teeth, but not for third molars [25].

The data used for the present study were cross-sectional. The oldest participants were born in the 1900s and the youngest in the 1960s. Appreciation of dentition, dental treatment strategies, and willingness to extract teeth have changed over these decades [8–10]. This is also evident in Figs. 1 and 2, where the youngest cohorts retained more teeth than older ones. An updated study on the most frequently extracted tooth is justified with current material to examine whether the preponderance of the third molar continues to hold up. It is anticipated that, along with improved dental health, extraction of teeth other than third molars will diminish.

The main limitation of this study was that the data were collected more than 20 years ago. Even so, the present analysis was carried out on this unique data with panoramic radiographs, as so far, no population-level studies on the most frequently extracted tooth have been published. Another limitation was the small number of remaining teeth in participants older than 90 years, which rendered most of the χ^2^ tests in that age group insignificant. Moreover, the data did not include information on congenital agenesis of individual teeth. However, the analysis took into account data on agenesis published in two meta-analyses [15, 16]. As the rates of agenesis are so low (0‒3.2%, third molars 22%), and the rates of missing teeth in the present study so high (16‒50%, third molars 53‒92%), the utilization of information from meta-analyses was acceptable. In addition, both meta-analyses on agenesis of teeth included a study also from Finland [26]. In that local study, the agenesis rate of third molars, 20.81%, falls within the 95% CI (18.4‒25.1) of Europeans in the first meta-analysis [15], which supports the comparability of values.

The main finding of the study is expected to be of use to clinicians and policy makers, but also to researchers writing forewords to their articles on third molars. The third molar, as the most common target for extraction, but also the most common tooth associated with malpractice claims [27], calls for skills, adequate equipment, and other resources for a successful extraction. Findings of the present study are applicable to populations in which impacted third molars cause inconvenience to individuals and are often extracted ahead of other tooth types.

In conclusion, based on this nationally representative sample of adults with panoramic radiographs, the most frequently extracted tooth was the third molar. Even considering the congenital agenesis of individual teeth, the third molar remained the most extracted tooth up to the age of 80 years.

## Data Availability

The data that support the findings of this study are available from the Finnish Institute for Health and Welfare. Restrictions apply to the availability of these data, which were used under license for this study. Data are available at http://www.terveys2011.info/aineisto/t2000/T2000_data.html with the permission of the THL Biobank.

## References

[1] Matzen LH, Schropp L, Spin-Neto R, Wenzel A (2017) Radiographic signs of pathology determining removal of an impacted mandibular third molar assessed in a panoramic image or CBCT. Dentomaxillofac Radiol 46:20160330. 10.1259/dmfr.20160330 27681861 10.1259/dmfr.20160330 PMC5595057

[2] Kautto A, Vehkalahti MM, Ventä I (2018) Age of patient at the extraction of the third molar. Int J Oral Maxillofac Surg 47:947–951. 10.1016/j.ijom.2018.03.020 29661639 10.1016/j.ijom.2018.03.020

[3] Sukegawa S, Yokota K, Kanno T, Manabe Y, Sukegawa-Takahashi Y, Masui M, Furuki Y (2019) What are the risk factors for postoperative infections of third molar extraction surgery: a retrospective clinical study. Med Oral Patol Oral Cir Bucal 24:e123–e129. 10.4317/medoral.22556 30573720 10.4317/medoral.22556 PMC6344007

[4] Sánchez-Torres A, Soler-Capdevila J, Ustrell-Barral M, Gay-Escoda C (2020) Patient, radiological, and operative factors associated with surgical difficulty in the extraction of third molars: a systematic review. Int J Oral Maxillofac Surg 49:655–665. 10.1016/j.ijom.2019.10.009 31735527 10.1016/j.ijom.2019.10.009

[5] Sifuentes-Cervantes JS, Carrillo-Morales F, Castro-Nunez J, Cunningham LL, Van Sickels JE (2021) Third molar surgery: past, present, and the future. Oral Surg Oral Med Oral Pathol Oral Radiol 132:523–531. 10.1016/j.oooo.2021.03.004 34030996 10.1016/j.oooo.2021.03.004

[6] Vranckx M, Fieuws S, Jacobs R, Politis C (2022) Surgical experience and patient morbidity after third molar removal. J Stomatol Oral Maxillofac Surg 123:297–302. 10.1016/j.jormas.2021.07.004 34260984 10.1016/j.jormas.2021.07.004

[7] Kempers S, van Lierop P, Hsu T-MH, Moin DA, Berge S, Ghaeminia H, Xi T, Vinayahalingam S (2023) Positional assessment of lower third molar and mandibular canal using explainable artificial intelligence. J Dentistry 133:104519. 10.1016/j.jdent.2023.104519 10.1016/j.jdent.2023.104519 37061117

[8] Centers for Disease Control and Prevention (2019) Oral Health Surveillance Report: Trends in Dental Caries and Sealants, Tooth Retention, and Edentulism, United States, 1999–2004 to 2011–2016. US Dept of Health and Human Services. https://www.cdc.gov/oral-health/php/data-research/2019-oral-health-surveillance-report/. Accessed 15 July 2024

[9] Jordan AR, Stark H, Nitschke I, Micheelis W, Schwendicke F (2021) Epidemiological trends, predictive factors, and projection of tooth loss in Germany 1997–2030: part I. missing teeth in adults and seniors. Clin Oral Investig 25:67–76. 10.1007/s00784-020-03266-9 33219875 10.1007/s00784-020-03266-9 PMC7785540

[10] Fuller E, Steele J, Watt R, Nuttall N (2011) 1: Oral health and function – a report from the Adult Dental Health Survey 2009. The NHS information Centre. https:/files.digital.nhs.uk/publicationimport/pub01xxx/pub01086/adul-dent-heal-surv-summ-them-the1-2009-rep3.pdf. Accessed 15 July 2024

[11] Information Services Division (2019) Scottish Adult Oral Health Survey 2016–2018. NHS National Services Scotland. https://www.scottishdental.org/wp-content/uploads/2019/04/2019-04-30-SAOHS-Report.pdf. Accessed 15 July 2024

[12] Suominen-Taipale L, Nordblad A, Vehkalahti M, Aromaa A (2008) Oral health in the Finnish adult population. Health 2000 Survey. Publications of the National Public Health Institute B25/2008. http://www.julkari.fi/handle/10024/103030. Accessed 15 July 2024

[13] Ventä I, Vehkalahti MM, Huumonen S, Suominen AL (2020) Prevalence of third molars determined by panoramic radiographs in a population-based survey of adult finns. Community Dent Oral Epidemiol 48:208–214. 10.1111/cdoe.12517 32003051 10.1111/cdoe.12517

[14] Hugoson A, Kugelberg CF (1988) The prevalence of third molars in a Swedish population. An epidemiological study. Community Dent Health 5:121–138 3165039

[15] Carter K, Worthington S (2015) Morphologic and demographic predictors of third molar agenesis: a systematic review and meta-analysis. J Dent Res 94:886–894. 10.1177/0022034515581644 25883107 10.1177/0022034515581644

[16] Polder BJ, Van’t Hof MA, Van der Linden FPGM, Kuijpers-Jagtman AM (2004) A meta-analysis of the prevalence of dental agenesis of permanent teeth. Community Dent Oral Epidemiol 32:217–226. 10.1111/j.1600-0528.2004.00158.x 15151692 10.1111/j.1600-0528.2004.00158.x

[17] Aromaa A, Koskinen S (2004) Health and functional capacity in Finland. Baseline results of the Health 2000 health examination survey. Publications of the National Public Health Institute B12/2004. http://www.julkari.fi/bitstream/handle/10024/78534/KTLB12-2004.pdf?sequence=1. Accessed 15 July 2024

[18] Heistaro S (2008) Methodology report. Health 2000 Survey. Publications of the National Public Health Institute B26/2008. http://www.julkari.fi/bitstream/handle/10024/78185/2008b26.pdf?sequence=1. Accessed 15 July 2024

[19] Koskela S, Vehkalahti MM, Suominen AL, Huumonen S, Ventä I (2022) Retained dental roots of adults: a nationwide population study with panoramic radiographs. Eur J Oral Sci 130:e12862. 10.1111/eos.1286 35363407 10.1111/eos.1286 PMC9324791

[20] Vehkalahti M, Ventä I, Valaste M (2023) Frequency and type of tooth extractions vary by age: nationwide observations from 2012 to 2017. Acta Odontol Scand 81:259–266. 10.1080/00016357.2022.2130977 36239127 10.1080/00016357.2022.2130977

[21] Eklund SA, Pittman JL (2001) Third-molar removal patterns in an insured population. J Am Dent Assoc 132:469–475 11315377 10.14219/jada.archive.2001.0209

[22] Magraw CBL, Moss KL, Fisher EL, Offenbacher S, White RP Jr (2016) Prevalence of visible third molars in the United States population: how many individuals have third molars? J Oral Maxillofac Surg 74:13–17. 10.1016/j.joms.2015.08.009 26355530 10.1016/j.joms.2015.08.009

[23] Jackson TH, Guez C, Lin F-C, Proffit WR, Ko C-C (2017) Extraction frequencies at a university orthodontic clinic in the 21st century: demographic and diagnostic factors affecting the likelihood of extraction. Am J Orthod Dentofac Orthop 151:456–462. 10.1016/j.ajodo.2016.08.021 10.1016/j.ajodo.2016.08.021 PMC5338460 28257729

[24] Bahrami G, Væth M, Kirkevang LL, Wenzel A, Isidor F (2008) Risk factors for tooth loss in an adult population: a radiographic study. J Clin Periodontol 35:1059–1065. 10.1111/j.1600-051X.2008.01328.x 19040583 10.1111/j.1600-051X.2008.01328.x

[25] Anagnou-Varelzides A, Komboli M, Tsami A, Mitsis F (1986) Pattern of tooth loss in a selected population in Greece. Community Dent Oral Epidemiol 14:349–352 3466764 10.1111/j.1600-0528.1986.tb01089.x

[26] Haavikko K (1971) Hypodontia of permanent teeth. An orthopantomographic study. Suom Hammaslääk Toim 67:219–225 5289906

[27] Koskela S, Suomalainen A, Apajalahti S, Ventä I (2017) Malpractice claims related to tooth extractions. Clin Oral Investig 21:519–522. 10.1007/s00784-016-1896-y 27511213 10.1007/s00784-016-1896-y

